# Educational Handoffs from Medical School to Residency: an Emerging Opportunity

**DOI:** 10.15694/mep.2017.000152

**Published:** 2017-08-30

**Authors:** Avraham Z. Cooper, Troy Schaffernocker, Jennifer McCallister

**Affiliations:** 1 Wexner Medical Center

**Keywords:** Milestones, Transitions in training, Competencies, EPAs

## Abstract

This article was migrated. The article was marked as recommended.

In this Personal View article we discuss the limitations of the summative medical student data currently received by residencies pre-match (such as transcripts, the Dean’s Letter and letters of recommendation) to adequately communicate a student’s strengths, weaknesses, and learning needs as they begin internship. We briefly summarize the evolution of medical student and resident performance evaluation, and discuss the role that educational handoffs may play in the future as students transition to internship. We then consider emerging questions about the feasibility and mechanics of educational handoffs and discuss possible steps forward.

## Background

Historically, residency program directors have received minimal (grades on a transcript), qualitative/descriptive, and non-standardized communication from medical schools prior to the match as part of the residency application process. The cornerstone of that communication is the Dean’s letter or medical student performance evaluation (MSPE), an often laudatory compilation of summative comments about a student’s strengths, weaknesses, and performance during medical school intended for use during the residency application process. Many have raised concerns about the utility and reliability of the MSPE to communicate students’ skills and educational needs anticipated during residency, purposes that it is not actually intended for [ Green, et al, 2012). Criticisms of the traditional Dean’s letter include that it is (a) not standardized, (b) susceptible to “code” language describing the quality of an applicant, (c) subjective, (d) labor intensive, (e) completed almost a year before internship, and (f) does not necessarily incorporate the large amounts of data currently collected regarding medical student performance [ Green, et al, 2012; Sozener, et al, 2016].

We suggest that neither the MSPE, the medical school transcript, nor letters of recommendation provide program directors with an adequate baseline on the clinical competence of their new trainees or what their learning needs will be. However, as assessment of medical students has become more standardized, there is an opportunity to provide granular, meaningful information at the transition between medical school and residency in the form of an educational handoff. We first discuss the current standards and literature of educational achievement monitoring at the graduate medical education (GME) level. We then explore the use of competency/milestone-based assessment and achievement in entrustable professional activities (EPAs) as potential reportable information, to be shared with residencies after the match but prior to the start of internship, to inform program directors of future training needs for residents. We then consider emerging questions about the feasibility and mechanics of educational handoffs and discuss possible steps forward.

## Evolution in performance assessment in graduate medical education

In 1999 the Accreditation Council for Graduate Medical Education (ACGME) introduced the six core competencies for resident and fellow performance, establishing a standard framework for training in GME, and eventually made documenting core competencies necessary for training program accreditation [ Swing, 2007]. In 2009 the ACGME further subdivided the assessment of the competency of residents and fellows into sub-competencies and milestones, which rated resident performance along a developmental continuum of achievement, and also allowed for specialty-specific metrics. Milestones facilitated tracking the progression of resident knowledge acquisition, attitudes, professionalism, and specialty-related skills at the level of individual programs and nationally [ Nasca, et al, 2012].

## Evolution in performance assessment in medical school

As graduate medical education assessment became more objective, a desire arose within undergraduate medical education to develop similarly quantitative outcomes, particularly for the transition from medical school to residency. In 2014 the AAMC introduced the Core Entrustable Professional Activities for Entering Residency (CEPAER) framework, which outlined thirteen activities (entrustable professional activities or EPAs) that all graduating medical students should be able to perform without direct supervision.). An individual EPA encompasses multiple skills and traits, subsumed under several different competencies and milestones. Examples include performing a history and physical, patient handoffs within transitions in care, or obtaining informed consent [ Aschenberger & Englander, 2014]. EPAs are increasingly utilized to evaluate students in the clinical years of medical school. This model has also been integrated into residencies for several specialties, including internal medicine and pediatrics [ Aylward, et al, 2014]. Similarly, to better prepare students for starting internship, medical schools have begun to incorporate competency and milestone-based assessment into clinical clerkships [ Khan, et al, 2016]. Many see this as the future standard of clerkship assessment.

## Emerging trends in educational handoffs

Competencies, milestones, and EPAs have emerged as potential components of an educational handoff to residencies. Milestone-based data collected in medical school, in particular, may be well-suited for this purpose as they offer a common language that can map directly to residency milestones. EPAs, because they are now routinely assessed during medical school and reflect practical skills that new interns should be able to perform independently, would provide program directors with a “functional baseline” of the abilities of their incoming residents.

Some medical schools have started to develop and assess a standard educational handoff to share with residency program directors. Sozener and colleagues at the University of Michigan piloted a milestone-based educational handoff for rising emergency medicine (EM) interns. Using milestone data collected toward the end of medical school in EM-related rotations to create performance evaluations, they shared these evaluations with several program directors, with positive preliminary responses regarding utility and usability [ Sozener, et al, 2016]. Wancata et al also at the University of Michigan, piloted a similar instrument with rising general surgery interns, mapping third and fourth year performance metrics to general surgery milestones. This too proved feasible, though program director satisfaction and feedback were not reported [ Sozener, et al, 2016]. Others have proposed an educational handoff system similar to handoffs in patient care, where medical schools summarize the competencies of graduating students, their performance, and any actionable items going forward [ Warm, et al, 2016]. As of 2016, the ACGME makes the residency milestone reports of incoming fellows available to fellowship program directors, to “ensure that the learner has an appropriate learning plan” as they start the next phase of their training [ Edgar & Holmboe, 2016]. A similar opportunity exists for improving the transition from student to resident.

## What comes next?

The ongoing evolutions in medical student and resident evaluation, away from the qualitative and descriptive and toward the quantitative and objective, reflect a larger trend in medical education of emphasizing educational outcomes over processes [ Chen, et al, 2004; Caraccio, et al, 2002]. The ultimate, short-term outcome at the medical school level is how a student performs as an intern and resident, and, indeed, concerns have been raised about the preparedness of rising interns in some specialties with respect to level-one milestone performance [ Santen, et al, 2013].

In the future, sharing competency, milestone, and EPA data between medical schools and residency program directors after the match, the performance of an educational handoff, could (a) establish an accurate baseline for evaluation at the level of internship and residency; (b) allow program directors to minimize redundant training of established skills; (c) readily establish a baseline for ACGME milestone tracking, if milestone achievement is incorporated into the handoff; (d) complement the formal testing of specific skills relevant to the trainee’s chosen specialty; (e) allow residency program directors to create a learning plan or adjust rotations at the start of internship for specific learner needs [ Lypson, et al, 2004].

Several questions about this process remain, meriting further investigation. Ongoing questions and possible next steps are listed below:

• What are the views of residency program directors, medical schools (e.g. deans, clerkship directors), and medical students about such sharing of information post-match?
  • National-level surveys of these groups would assess how they feel about educational handoffs and help guide policy decisions going forward
• What would the mechanics of information sharing entail, especially since medical schools are not necessarily measuring milestones, competencies and EPAs directly relevant to a specific specialty, or at all [ Franzen, et al, 2015]?
  • Feasibility, pragmatic studies of potential handoff templates across multiple specialties would outline how well different formats work in real-world milieu (e.g. ease-of-use, applicability, which metrics were felt to be most relevant to residency programs)
• How predictive of residency performance are medical school EPAs, competencies, and milestones?
  • A study mapping these metrics to their residency equivalent (particularly for milestones) or correlating with other markers of performance in residency would better define what information types are best-suited for use in handoffs to residencies

## Conclusions

More research is needed to both identify the attitudes of the stakeholders in this process, and establish optimal mechanisms and timing, before medical school-to-residency educational handoffs are implemented nationwide. While it seems likely, even inevitable, that within 5-10 years medical schools will routinely perform educational handoffs at graduation, how we get there and what it will look like remain unknown.

## Take Home Messages

• The summative medical student data currently received by residencies may not adequately communicate a student’s strengths, weaknesses, and learning needs as they begin internship
• Educational handoffs likely will play a future role in facilitating the transition from student to resident, though questions remain about feasibility and the mechanics of this process

## Notes On Contributors

Dr. Cooper is a pulmonary/critical care fellow in the Division of Pulmonary, Critical Care & Sleep Medicine at The Ohio State University, Columbus, OH.

Dr. Schaffernocker is an Assistant Clinical Professor in the Division of Pulmonary, Critical Care & Sleep Medicine at The Ohio State University, Columbus, OH.

Dr. McCallister is an Associate Clinical Professor in the Division of Pulmonary, Critical Care & Sleep Medicine and program director of the Pulmonary/Critical Care Fellowship at The Ohio State University, Columbus, OH.
