# Supplementary material for: Educational Handoffs from Medical School to Residency: an Emerging Opportunity
Source: MedEdPublish (2016). 2017 Aug 30;6:152. [Version 1] doi: 10.15694/mep.2017.000152 (PMC10885287; doi:10.15694/mep.2017.000152)
Supplement: Peer review history

# Reviewer report: Reviewer response for version 1

**DOI:** 10.21956/mep.19339.r28248

**Published:** 2017-10-17

**Authors:** Julie Hunt

**Affiliations:** 1 Lincoln Memorial University

This review has been migrated. The reviewer awarded 4 stars out of 5 This opinion piece describes the current US situation of educational handoffs and highlights how this system could be enhanced. The issue that the authors raise is timely and real. The authors provide a list of pertinent questions raised and identify potential areas for subsequent research which may be helpful to the field.

Reviewer Expertise:

NA

No decision status is available

# Reviewer report: Reviewer response for version 1

**DOI:** 10.21956/mep.19339.r28247

**Published:** 2017-09-10

**Authors:** Trevor Gibbs

**Affiliations:** 1 AMEE

This review has been migrated. The reviewer awarded 3 stars out of 5 The transition from medical student, through whatever form of internship through to a competent practitioner capable of working effectively and efficiently without supervision is a difficult period and as the authors suggest, one that merits more research. The introduction of another set of potentially achievable outcomes is one way to achieve a degree of assurance that the practitioner is indeed competent. These educational handoffs are / can be effective in assessing the student in the real world, providing the outcomes have been clearly defined. This paper is US-centric and is probably effective in a school that has set out clear outcomes for graduation and for different levels of postgraduate development.- I wonder how it would fare in less-developed programmes, in less developed countries, where that clarity is less certain and where the drive is to graduate as many practitioners as possible for an over-burdened population.However, I did think that the paper was a worthy read and, as papers like this should, it raises as many questions as it has answered

Reviewer Expertise:

NA

No decision status is available
